# Knudsen pumps: a review

**DOI:** 10.1038/s41378-020-0135-5

**Published:** 2020-06-15

**Authors:** Xiaowei Wang, Tianyi Su, Wenqing Zhang, Zhijun Zhang, Shiwei Zhang

**Affiliations:** School of Mechanical Engineering and Automation, Northeastern University, 110819 Shenyang, China

**Keywords:** NEMS, Structural properties

## Abstract

The Knudsen pump (KP) is a kind of micro-pump that can form thermally induced flows induced by temperature fields in rarefied gas environments. It has the advantages of having no moving parts, simple structure, easy construction and extension, a wide range of energy sources, and low energy consumption. With the development of Micro/Nano Electro Mechanical Systems (MEMS/NEMS), extensive studies have been conducted on KPs, and the applications of KPs have widened. In order to obtain efficient flow fields in KPs, it is necessary to adopt modern computational methods for simulation and analysis. In many circumstances, the simulation and experimental results have good agreement. However, there seems to be no comprehensive review on KPs at present. In this paper, KPs are first defined and classified according to the flow mechanisms of the thermally induced flows. Then, the three aspects of configurations, performance, and applications of KPs in the current state of research are reviewed and analyzed. Finally, the current problems of KP are discussed, and some suggestions are provided for future research and applications.

## A review of Knudsen pumps: Gas-pumps with no moving parts

Knudsen pumps are micro-sized, non-mechanical gas pumps that can be useful in a range of biomedical and mechanical fields. Zhang and his team from China’s North-eastern University analyzed Knudsen pumps’ configurations, performance, applications, and current problems. In Knudsen pumps, gas flow is induced by temperature gradients along the walls of the device. They benefit from low energy consumption and ease of manufacture. Different configurations, realized through the placement of variably heated walls, offer multiple mechanisms to transport rarefied gases. Knudsen pumps can be used, amongst other applications, to generate a vacuum, deliver fluids, and separate gases. Micro-channels play a key role in Knudsen pump efficiency and gas flow operation; however, there is a lack of research into efficiency which the authors say should be addressed in future research.

## Graphical abstract

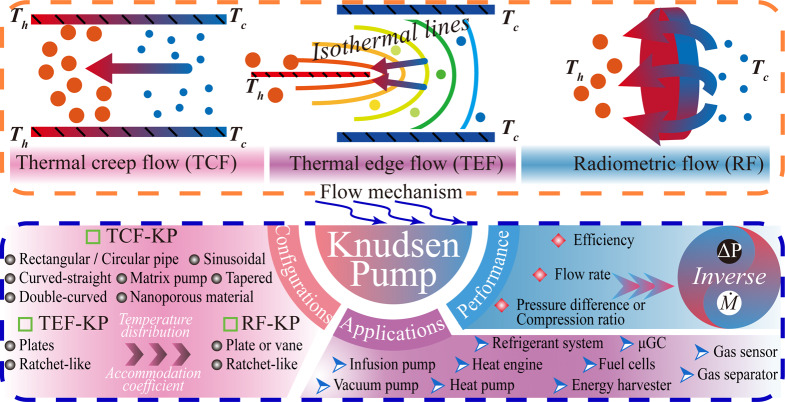

## Introduction

Micro electro mechanical systems (MEMS) were first introduced in the 1990s^1^. They have since been widely used in many fields due to their large quantity production, high integration, easy extension, and interdisciplinary applications. Research has been conducted on various microfluidic devices, such as micro fuel cells^2–4^, lab-on-a-chip systems^5^, micro-gas chromatographs^6–10^, micro mass spectrometers^11–13^, micro spectrometers^14,15^, reformers^16–18^, and micro air vehicles^19–23^. The micro-pump, which is a driven control engine for micro fluids, is the key to the development of microfluidic devices, and is a major area of research.

Micro-pumps exist with various actuating principles and morphologies. There are mechanical micro-pumps and nonmechanical micro-pumps, classified according to whether they have moving parts. Mechanical micro-pumps use the mechanical energy of their moving parts to drive fluid flow, and their working mediums are basically liquids. Their life spans, sensitivity, and stability are not good due to the moving parts. In contrast, fluid flow in nonmechanical micro-pumps is induced by nonmechanical forms of energy such as electric energy, thermal energy, chemical energy, and magnetic energy. Their working mediums are not limited to liquids but can also include gases or solids in the form of nanometer-sized particles.

Mechanical micro-pumps can be classified into various types including piezoelectric pumps^24,25^, electrostatic pumps^26,27^, thermo pneumatic pumps^28^, shape-memory alloy (SMA) pumps^29^, and electromagnetic pumps^30^ based on the type of actuating force. Nonmechanical micro-pumps can be divided into categories that include magnetohydrodynamic pumps^31^, electroosmotic pumps^32^, and Knudsen pumps (KP)^33,34^. Due to their different mechanisms, micro-pumps and non-micro-pumps differ from each other. Pumps can also be classified based on their applications. In MEMS, nonmechanical micro-pumps generally have better performance and more favorable research and development prospects compared to mechanical micro-pumps. Since the first use of piezoelectric pumps in an insulin transport system in 1990^35^, a large number of research achievements have been reported and well reviewed elsewhere^36,37^.

A nonmechanical micro-pump where the gas flow is induced by temperature gradients along the walls was first proposed by Knudsen^38,39^. Such micro-pumps were later called KPs or Knudsen compressors. The working mechanism of the KP is based on the thermal creep effect in rarefied gases^40,41^. KPs have always received much attention from many scholars due to their advantages of having no moving parts, simple structures, wide variety of energy sources, and low energy consumption, and their easy mass production and extension. Moreover, with the development of micro-manufacture technologies^42^ and the progress in material analysis and computational methods, both the applications and theories of KPs have developed unprecedentedly. However, a systemic analysis and summary of the current research achievements in KPs is still lacking.

To address this gap in the literature, this paper aims to provide relevant information on KPs. The flow mechanisms of thermally induced flows in rarified gases and the classification of KPs are presented in the section “Flow mechanisms of thermally induced flow and classifications of KPs”. The current configurations of KP based on different methods to thermally induce flows and the computational methods for research are discussed in the section “Configurations of KPs and computational approaches”. The state of research on the performance of KPs is briefly reviewed in the section “KP performance research”. Research on the applications of KP is discussed in the section “Research on KP applications”. In the section “Discussions and outlook”, some perspectives on the future development of KPs are put forward and discussed. The relevant conclusions are presented in the section “Conclusions”.

## Flow mechanisms of thermally induced flow and classifications of KPs

### Flow mechanisms of thermally induced flow

The degree of gas rarefaction is determined by the Knudsen number (*Kn*), which is defined as the ratio of the molecular mean free path *λ* to the characteristic flow length scale *L*,

1 $$Kn = \frac{\lambda }{L}$$

Based on the value of *Kn*, the flow stages of gases can be classified into inviscid flow (*Kn* → 0), the continuum regime (*Kn* ≤ 0.001), the slip regime (0.001 ≤ *Kn* ≤ 0.1), the transition regime (0.1 ≤ *Kn* ≤ 10), and the free-molecular regime (10 ≤ *Kn*)^43,44^. Note that this classification scheme is based on isothermal gas flow. The continuum regime is dominated by collisions between gas molecules. The gas is in perfect thermodynamic equilibrium (isothermal gas flow), especially when *Kn* → 0. With the increase of *Kn*(0.001 ≤ *Kn*), molecular collisions and molecule−surface interactions become less frequent, and the nonequilibrium regime begins to appear near the surfaces. This phenomenon leads to differences between the velocity and temperature of the gas at the surface and of the surface itself, i.e., velocity slips and temperature jumps appear between the gas and the surface. In the transition and free-molecular regimes, nonequilibrium effects dominate, especially when 10 ≤ *Kn*, and collisions between molecules become negligible.

**Fig. 1:**
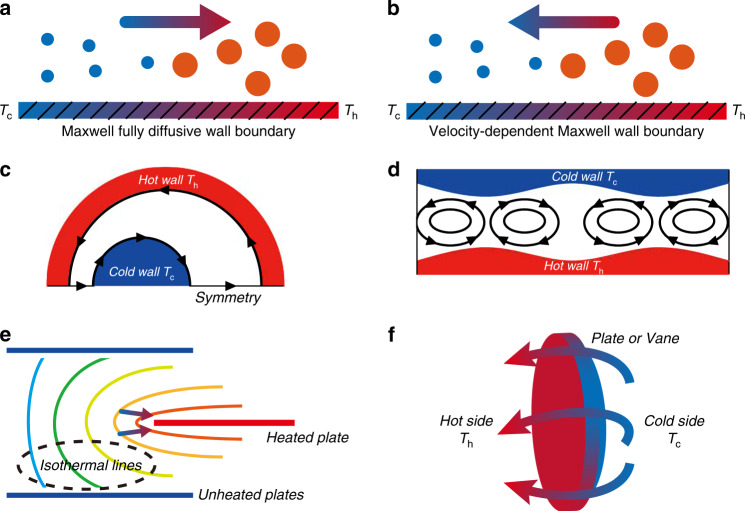
Classification of thermally induced flows. **a** Thermal creep flow, **b** inverted thermal creep flow, **c** thermal stress slip flow, **d** (nonlinear) thermal stress flow, **e** thermal edge flow, **f** radiometric flow

In the framework of classical fluid dynamics, there is no time-independent flow induced by virtue of only the temperature field without external forces such as gravity and pressure. However, in a rarefied gas (0.001 ≤ *Kn*), the temperature field plays an important role in inducing time-independent flow. According to the asymptotic theory for small *Kn* and numerical observations based on kinetic theory^45,46^, the gas flow induced by the temperature field can be classified as follows: thermal creep flow (Fig. 1a)^40,41^, inverted thermal creep flow (Fig. 1b)^47,48^, thermal stress slip flow (Fig. 1c)^49,50^, (nonlinear) thermal stress flow (Fig. 1d)^51–53^, thermal edge flow (Fig. 1e)^54,55^, radiometric flow (Fig. 1f)^56,57^, and thermophoresis^58^. Note that the motion of particles in a gas with a temperature gradient is called thermophoresis. This phenomenon involves solid particles and is not included in the review. The other flows and their mechanisms will be analyzed in the following sections.

#### Thermal creep flow

The theories and applications of the thermal creep effect seem to be the richest among the various types of thermally induced flows. Research on the thermal creep effect can be dated back to the nineteenth century^40,41^. A typical application of thermal creep flow is the Knudsen pump. Moreover, the thermal creep flow is the main working mechanism for the Hettner radiometer^59^. In space, thermal creep flow is used to explain the phenomena of protoplanetary disks and gas, and the eruptions of ash layer particles on the surface of Mars^60–65^. What is more notable is that the motion of solids^61^ and the recurring slope line (RSL)^66^ on the surface of Mars are probably triggered by thermal creep flow.

**Fig. 2:**
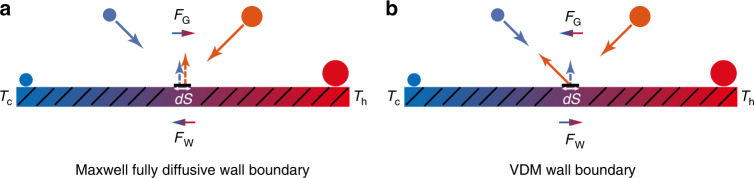
Schematics of two gas flow mechanisms. **a** Thermal creep flow, **b** inverted thermal creep flow. The sizes of the circular symbols depict the gas velocity, and their colors depict the gas temperature

To explain the physical mechanism of thermal creep flow, we consider the static gas on a wall with a temperature gradient as shown in Fig. 2a. Take a small area *dS* of the wall, and consider the momentum transferred to *dS* when gas molecules impinge on it. The molecules impinging on *dS* have a distance on the order of the mean free path. Since the molecules coming from the hotter region have a larger average speed than the molecules coming from the colder region, the momentum transferred to *dS* (or wall) by the molecules impinging on it has a tangential component in the direction opposite to the temperature gradient. More precisely, in a static gas, the number of molecules in the colder region is larger than that in the hotter region (the molecular density in the hotter region is smaller); the molecules from these two regions impinging on *dS* per unit time are the same^45,46^. The molecules from the hotter region have a larger average speed. Thus, the momentum transferred to *dS* by the molecules impinging on it has a tangential component in the direction opposite to the temperature gradient. Note that for the diffuse reflection, the contribution of the molecules leaving the wall to the tangential component of the momentum transfer is highly small (almost zero)^45,46^. Summarizing, a net force (*F*_W_ in Fig. 2a) towards the colder region is produced by the molecules on the wall. Therefore, the gas is subjected to a reaction force acting in the direction of the temperature gradient (*F*_G_ in Fig. 2a) according to Newton’s third law, and a gas flow results in this direction. Note that modern numerical analysis^67,68^ has discovered that the force direction on the wall with a temperature gradient is the same as the gas flow direction. The probable explanation for this is that in the moving gas, the momentum in the direction of the motion is transferred to the wall^46^.

From the above illustrations, it is easy to see that the boundary (usually assumed as Maxwell fully diffusive wall boundary) plays a crucial role in inducing thermal creep flow. However, if the gas molecules impinge on a VDM (velocity dependent Maxwell) wall boundary, inverted thermal creep flow will be produced (Fig. 2b) with a flow direction opposite to that of thermal creep flow^47,48^. The reason for the inverted flow is that on the VDM boundary, the gas−surface interaction from the impingement of molecules on *dS* (or wall) is related to the velocities of the molecules^47,48^. As a result, a net force towards the hotter region is imparted on the wall (*F*_W_ in Fig. 2b). In turn, the gas adjacent to the wall is subjected to a reaction force from the wall and driven toward the colder region (*F*_G_ in Fig. 2b). This causes the movement of the gas from the hotter region to the colder region of the wall. Note that the inverted thermal creep is only observed theoretically, has not yet been verified in experiments.

#### Thermal stress slip flow and (nonlinear) thermal stress flow

**Fig. 3:**
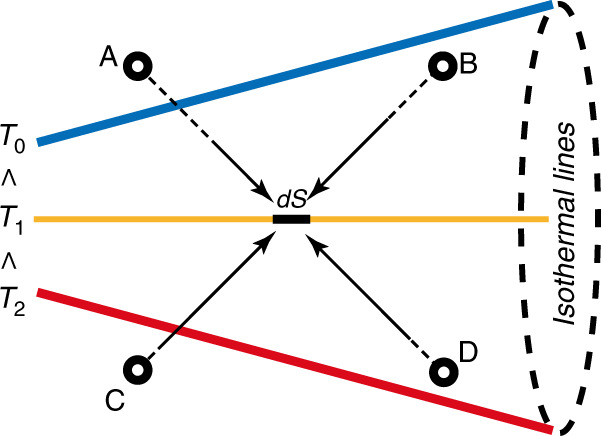
Source of thermal stress

Based on the above illustrations, there is no thermal creep flow or inverted thermal creep flow induced over a simple boundary with a uniform temperature. Thermal stress flow^45,46^ is triggered when the temperature gradient normal to the boundary is not uniform over the boundary, or when the isothermal surface is not parallel to the boundary. This flow occurs in the direction where the isothermal surfaces diverge or converge according to whether the boundary temperature is lower or higher than that of the surrounding gas. In order to illustrate the reasons for the appearance of thermal stress slip flow, the source of thermal stress is first analyzed. A small area *dS* is taken in a gas with inhomogeneous temperature gradient, shown as Fig. 3. The four dots A, B, C and D (the distance between *dS* is about one mean free path) are used to represent the molecules from different directions impinging on *dS*. The different initial temperatures of these molecules (A, B, C, and D) result in different momentums transferred by *dS* (the higher the initial temperature is, the larger the mean velocity is). Thus, thermal stress occurs over *dS*^45,46^.

**Fig. 4:**
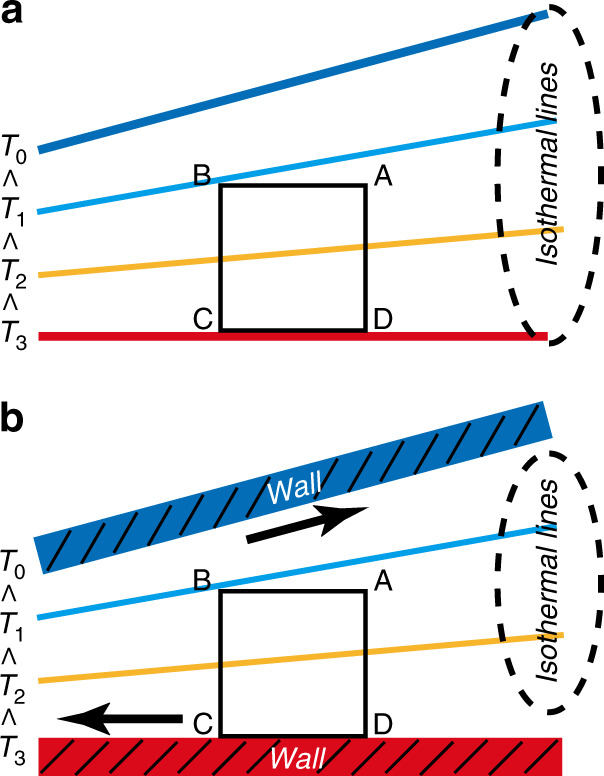
Schematic of thermal stress slip flow mechanism. **a** The control surface is in the gas and **b** the part CD of the control surface is on a wall

Now, how the thermal stress slip flow is induced by thermal stress is considered. A control surface ABCD is taken in a gas with a nonuniform temperature gradient. One of the surfaces (CD) is located on an isothermal surface as shown in Fig. 4. When the control surface is in a nonboundary gas (Fig. 4a), the thermal stress resulting from energy transfer due to temperature variation balances by itself in a closed surface (or a control surface) so that the net force is zero^45,46^. Thus, thermal stress cannot cause the gas to flow. However, the situation will differ if the gas is surrounded by two walls with uniform temperature as shown in Fig. 4b. In this case, the thermal stresses on the surfaces AB, BC, and AD are almost the same as before in the nonboundary gas presented in Fig. 4a, but the momentum transfer from the molecules impinging on CD (wall) is about half of the overall transfer in the nonboundary gas^45,46^. That is because when surface CD is in a nonboundary gas (or on the wall), the molecules impinging on CD with momentum transfer correspond to the molecules from A, B, C and D in the preceding paragraph (or molecules from A and B). Therefore, due to the boundary wall CD, the balance of the thermal stress on the control surface ABCD is violated. This triggers the thermal stress slip flow in the direction indicated by the black arrows in Fig. 4b.

It is noted that similar to the thermal creep flow and the inverted thermal creep flow, the boundary walls play an important role in inducing thermal stress slip flow. Nevertheless, there is another flow, (nonlinear) thermal stress flow^52^, induced by boundary walls indirectly which is different from the above flows. More precisely, the thermal stress tensor produced in a gas with a large temperature variation includes linear terms in the temperature change and nonlinear terms, which cannot be neglected for a large temperature variation. Nonlinear thermal stress on a closed surface (control surface) does not balance itself in a nonboundary gas^45,46^. In contrast, linear thermal stress balances over a closed surface in a nonboundary gas as mentioned in the last section. Therefore, if the isothermal surfaces are not parallel (nonuniform temperature gradient), nonlinear thermal stress flow will occur.

Note that the flow pattern (including the flow direction) of this nonlinear thermal stress flow is highly similar to that of the thermal stress slip flow. The differences of the flow structures lie in that nonlinear thermal stress flow occurs in a gas in which the temperature gradient is nonuniform, whereas thermal stress slip flow appears over a wall boundary with a nonuniform temperature gradient^49,69^. By the way, detailed mathematical analysis by Sone^45,46^ showed that the thermal stress flow and thermal creep flow are proportional to the first order in the temperature field derivatives, but thermal stress slip flow is proportional to the second order in the temperature field derivatives. Moreover, over walls with temperature gradients, thermal stress slip flow is usually overshadowed by thermal creep flow.

#### Thermal edge flow and radiometric flow

For the flows discussed above, the shapes of the boundaries are all assumed to be smooth without sharp edges. In fact, sharp curves on the isothermal surfaces in the vicinity of the surface edges can trigger another two types of thermal flow, namely, the thermal edge flow and the radiometric flow.

**Fig. 5:**
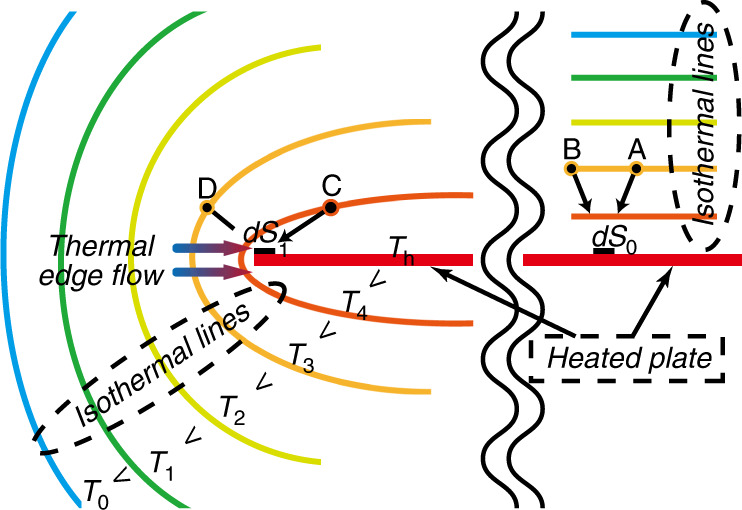
Schematic of thermal edge flow mechanism

The thermal edge flow was first discovered by Aoki et al.^54^ and then experimentally verified by Sone and Yoshimoto^55^. Consider a uniformly heated plate in a gas with sharp boundary edges and a size much larger than the mean free path of the gas shown in Fig. 5. Evidently, the isothermal surfaces near the edge of the plate curve sharply, leading to gas flow. In contrast, the isothermal surfaces far away from the edges are parallel to the plate and thus no gas flow occurs^45,46^. To be precise, the momentum transfer from the molecules (represented by the four points A, B, C, and D around one mean free path away from the impingement point) impinging on the plate are of two kinds, zero momentum exchange and finite momentum exchange. Due to symmetry, we consider two regions far away from the edge *dS*_0_ and *dS*_1_ on only the upper side of the plate. When the molecules A and B impinge on *dS*_0_, there is no tangential momentum exchange since they are from the same isothermal surface. However, when the molecules C and D impinge on *dS*_1_, molecule C from the hotter region has a larger mean velocity than molecule D from the colder region, and thus a component of the momentum toward the edge of the plate is transferred to *dS*_1_. That is to say, a net force toward the colder region is produced on the surface edges. Therefore, a net reaction force on the gas is produced toward the hotter region, and a gas flow indicated by the colorful arrows in Fig. 5 is induced in this direction. It is apparent that the mechanism of the flow is similar to the thermal creep flow over walls with temperature gradients. Note that the value of the thermal edge flow proportional to the 0.5th order in the temperature field derivatives^46,55^.

**Fig. 6:**
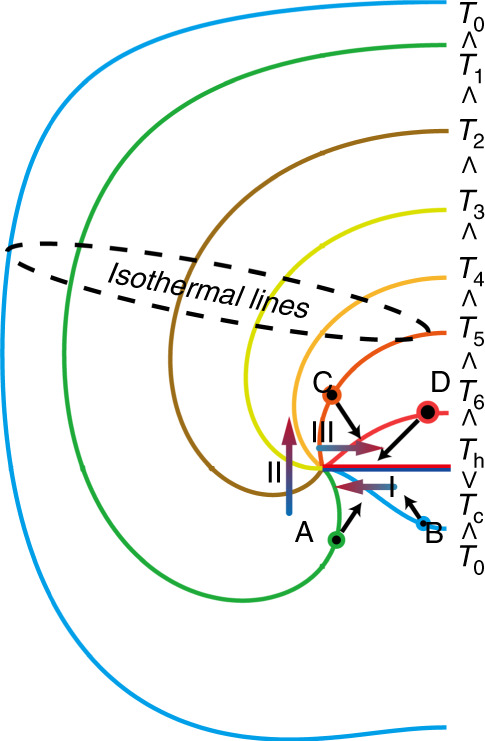
Schematic of radiometric flow mechanism

In the discussion above, we have assumed that the temperature of the plate is homogeneous, and the molecules over the two sides of the plate are symmetrically distributed and have the same temperature. Thus when the molecules impinge on the plate, there is no net momentum exchange in the direction normal to the plate. However, if the temperatures of the two sides of the plate are different, a different situation will occur and radiometric flow will be generated^56,57^. Consider a plate placed in a gas. The right side of the plate is heated uniformly and the left side is not heated, as shown in Fig. 6. Under these circumstances, the temperature decreases towards the edge along the heated side of the plate, but increases towards the edge along the unheated side. Through a similar mechanism to the inducing of thermal creep flow, this kind of temperature field distribution will lead to the generation of two reverse induced flows in which the gas on the unheated side flows towards the edge of the plate and the gas on the heated side flows towards the center of the plate. This means that the gases flow from the unheated (colder) to the heated (hotter) side along the plate, as indicated by the colorful arrows in Fig. 6.

More precisely, the molecules impinging on the two sides of the plate are represented by four points A, B, C, and D at about one mean free path away from the impingement point. On the unheated side, since the momentum transfer from the impingement of A from the hotter region is larger than that from the impingement of B from the colder region, a net momentum component towards the center of the plate is transferred. In other words, a net force acts towards the center of the plate. This means that the gas is subjected to a reaction force towards the edge of the plate, which causes a flow in this direction (indicated by the colorful arrow I in Fig. 6). On the heated side, a similar flow is also generated by the impingement on the plate. Since the temperature of the molecule D is higher than that of C, the flow direction is towards the center of the plate as presented by the colorful arrow III in Fig. 6. Therefore, a gas flow, shown as the colorful arrows I, II, and III in Fig. 6, is generated from the unheated side to the heated side.

It is worth noting that the flow patterns of the thermal edge flow and radiometric flow are similar at large *Kn*. However, their flow patterns have significant differences at small *Kn*^56,57^. In summary, the physical mechanism for inducing radiometric flow is similar to the mechanisms for inducing thermal creep flow over walls with temperature gradients and the appearing of the thermal edge flow over the sharp edges of a uniformly heated plate. In particular, the flow directions of these three flows are all from the colder region towards the hotter region, which makes the gas pumping of KPs possible.

### Classification of KPs

The first application of thermal creep flow is probably the KP^38,39^, and the initial applications of thermal edge flow and radiometric flow are probably the Nichols^70^ and Crookes radiometers^71,72^, respectively. With the further development of high-performance micro-devices, the two types of radiometers are widely used in sensors, atomic force microscopes (AFMs), and propulsion systems^73,74^. A review^68^ reports the progress in research on radiometers from the end of the nineteenth century to the beginning of the twenty-first century in detail. Note that the review focused on research on the forces on solid surfaces resulting from thermal creep flow, thermal edge flow, and radiometric flow. However, researching the gas flow in nanometer- and micron-sized channels has significant practical and scientific value^75^. Recent experimental and numerical works^56,76,77^ indicate that thermal edge flow and radiometric flow can also be used in KPs. Based on the above discussion on thermally induced flows, KPs can be classified into thermal creep flow KPs, thermal edge flow KPs, and radiometric flow KPs based on the dominant flow in the KP.

#### Thermal creep flow KP

**Fig. 7:**
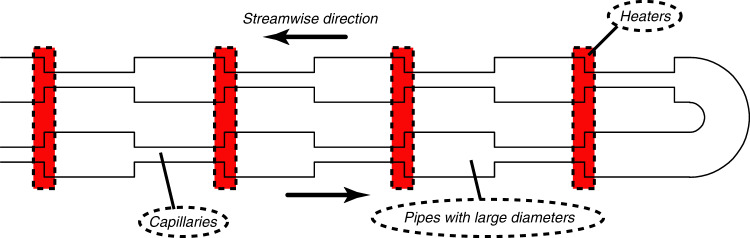
The model of an early thermal creep flow KP

Thermal creep flow KPs are the first type of KPs to be proposed. The model constructed by Knudsen^38,39^ is shown in Fig. 7. It consists of an alternating series of large-diameter pipes and capillaries where heaters are placed on one end of the pipes with large diameters. If the diameters of the capillaries are small enough compared to the molecular mean free path of the gas, the rarefied gas condition can be satisfied. Using the heaters to heat one end of the capillaries, the thermal creep effect is induced by the temperature gradients parallel to the walls, which drive gas flow from the cold sides to the hot sides. In contrast, the thermal creep effect is negligible in the large-diameter pipes due to the much bigger sizes of the pipes compared to the gas molecular mean free path. The gas flow is hence not significantly hindered.

As more and more gas molecules gather on the hot side under the influence of temperature, the pressure difference between the cold and hot sides increases. Driven by the pressure difference, Poiseuille flow appears. Thus, part of the gas on the hot side flows again to the cold side, weakening the flow due to the thermal creep effect. The combined action of the two flows ultimately puts the KP into dynamic equilibrium and the net mass flow rate is zero. The theoretical relation between the high−low pressure of the two sides and the temperature can be represented as^78,79^

2 $$P_{{\mathrm {low}}}/P_{{\mathrm {high}}} = (T_{\mathrm c}/T_{\mathrm h})^{\frac{n}{2}}$$

where *n* is the cascade stage number, and *P*_low_ and *P*_high_ are the pressures on the low- and high-pressure sides, respectively. *T*_h_ and *T*_c_ are the temperatures on the hot and cold sides, respectively. With the increase of *n*, the pressure difference increases gradually to achieve the function of a vacuum pump. The performance and applications of such pumps will be discussed in the following chapters.

#### Thermal edge flow KP

**Fig. 8:**
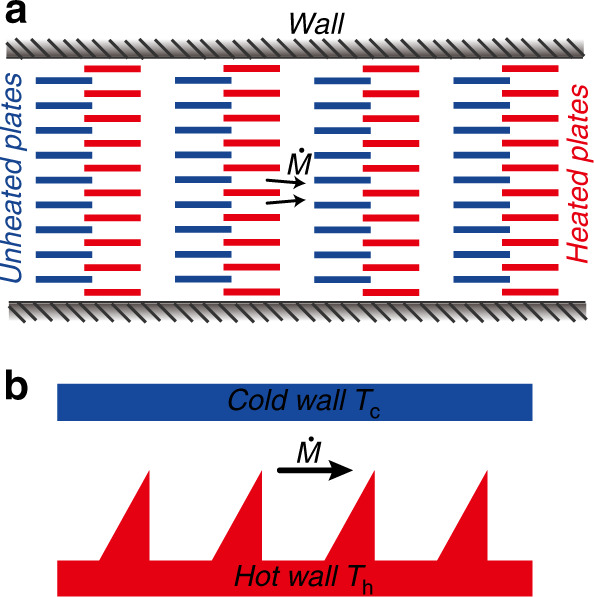
Model of thermal edge flow KP

Although the thermal edge flow phenomenon has a long history, it was only in the 1990s^53–55^ that a thermal edge flow KP was first assembled and tested^76^. The pump is constructed by inserting parallel heated and unheated plates in a steel pipe, as shown in Fig. 8a. In fact, besides plates with ratchet edges, thermal edge flow can also be triggered on plates with smooth edges^53^. If the structure surface has sharp corners as shown in Fig. 8b, the temperature fields in the vicinity of the corners change more dramatically and thermal edge flow is enhanced. Thermal edge flows can be formed on constant temperature walls, eliminating the need for temperature gradients necessary in thermal creep flow KPs. This substantially reduces the requirements for temperature control and therefore makes thermal edge flow KPs easier to apply in practice.

#### Radiometric flow KP

**Fig. 9:**
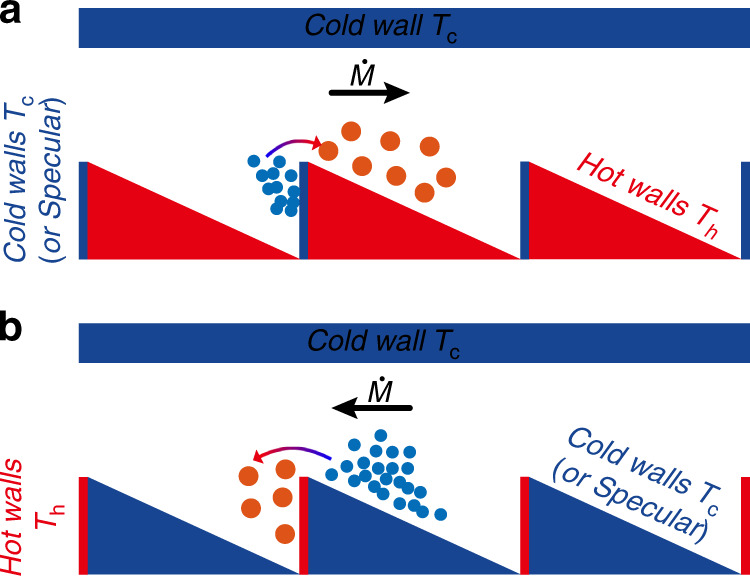
Model of radiometric flow KP

In the above discussions, it is assumed that the surface accommodation coefficients of all the walls are the same (diffuse reflection walls). However, radiometric flow KPs can be easily obtained by changing the accommodation coefficients of the adjacent walls in thermal edge flow KPs^80,81^. Moreover, radiometric flow KPs can also be obtained through adjusting the temperature of the adjacent walls in thermal edge flow KPs^82^, as shown in Fig. 9. In fact, the radiometric flow KP can be seen as a development of the thermal edge flow KP. Regardless of whether the accommodation coefficient or the temperature is changed, it is the thermalizing process of the gas molecules colliding with the walls that is affected. The gas molecules have weak or zero thermalization when they collide with cold or specular surfaces. In contrast, gas molecules are strongly thermalized with the surfaces when they collide with the hot or diffuse walls. As a result, gas flow occurs from the cold side to the hot side. Note that if the cold or specular walls are inclined, radiometric flow becomes more significant. Although the Crookes radiometer^71,72,83^ is one application of radiometric flow, research on radiometric flow KPs is still in the stage of theoretical analysis and numerical simulation. More research and experiments are required to confirm the actual behaviors of radiometric flow KPs.

## Configurations of KPs and computational approaches

### Thermal creep flow KP configuration

In the early twentieth century, Knudsen constructed a thermal creep flow KP system^38,39^, conducted experiments on the system, and verified its feasibility. KPs have since consistently attracted the attention of many scholars. In a typical thermal creep flow KP^38,39,84^, two cavities with different temperatures are connected by a circular cross-section microtube. Various varieties of thermal creep flow KPs are currently being studied^85–88^ as a result of the greatly enriched set of methods and approaches for obtaining micro-channels due to the rapid development of micromachining and material techniques.

Generally, any shape of micro-channel can be obtained by applying selective etching and micromachining techniques. However, the micro-channels are usually manufactured as rectangles^33,34,79,89–93^ on one hand due to the relatively low manufacturing complexity, and on the other hand due to the high performance of rectangular shaped thermal creep flow KPs^94^. A vertically stacked thermal creep flow KP was manufactured in 2013 using micromachining techniques.^34^ This kind of KP overturns the traditional planar arrayed layout^34^ and is a good choice for applications in limited space. However, multistage cascading is detrimental to heat dissipation, resulting in decreased temperature difference and degraded performance compared to the planar arrayed layout.

Many kinds of nanometer porous materials, both natural and artificially synthesized, are used for the manufacture of thermal creep KPs. These materials must satisfy several requirements. First of all, the hole diameters of the materials must ensure that the gas flow is in the transitional or free-molecular state. Secondly, in order to induce thermal creep flow successfully, sufficiently large temperature gradients must be established in the porous materials, i.e., the thermal conductivity of the porous materials should be low. At present, a number of porous materials for thermal creep flow KPs have already been assembled and tested. These materials are namely, aerogel membranes^95–98^, mixed cellulose ester (MCE)^99–105^, natural zeolite^106,107^, porous ceramics^108,109^, glass^110,111^, and Bi_2_Te_3_^112^. Note that in most of these KPs, a heater is put on one side of the nanometer porous material to increase the temperature and a heat sink is put on the other side to lower the temperature in order to obtain a larger temperature difference. However, in KPs in which thermoelectric materials are used^99^, the thermoelectric materials serve as both heater and heat sink to increase the temperature difference between the two sides of the nanometer porous material. In addition, the positions of the cold and hot sides of the nanometer porous materials can be switched by reversing the electric current direction to realize two-way working of the KPs. However, due to the air gaps between the heater, heat sink, thermoelectric material, and nanometer porous material, the maximum achievable temperature difference between the two sides of the micro-channels is limited and hence the pump performance is impeded. Constructing the KP^112^ out of multifunctional nanometer Bi_2_Te_3_ thermoelectric material significantly improves the pump performance. The submicrometer holes in the material serve as channels for gas flow. The thermoelectric character of the materials is used to establish the temperature difference for inducing thermal creep flow. Moreover, if an extra heat source is provided, the KP can possibly generate electric power^112^. Please note that this conjecture has not been demonstrated theoretically or experimentally. If the conjecture is true, the KP can be used as an electric generator.

In order to quantify the gas flow patterns in KPs, tracer particles are usually used to visualize the flow^88,111^. However, with the development of computer technology, numerical simulations can be used to show the gas flow patterns more directly. Numerical simulations of thermal creep flow in porous structures^113–115^ are important in guiding the design and optimization of KPs. In addition, numerical simulation is employed in many research works on variations of thermal creep flow KPs due to the high efficiency, completeness, and economy of numerical simulation.

**Fig. 10:**
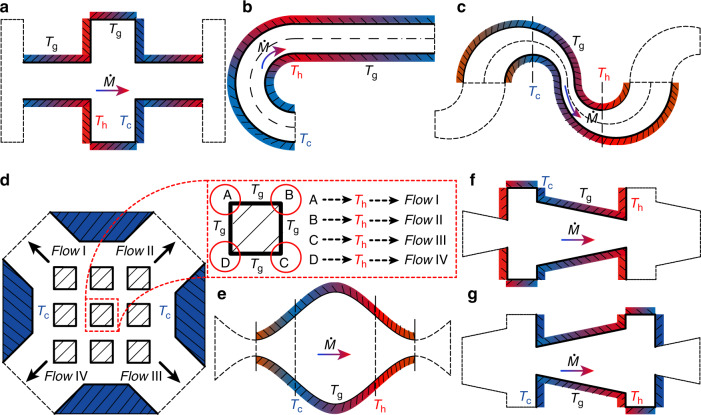
Thermal creep flow KP channel structures and configurations

When thermal creep flow KPs are modeled numerically, calculations are usually performed on only parts of the channel due to the periodicity of the channel structure. A cylindrical channel can reflect the actual thermal creep flow phenomenon in a pump^116,117^. As a three-dimensional simulation will substantially increase the cost, the numerical calculation is usually limited to the two-dimensional plane. The thermal creep flow between parallel plates is usually the simplest problem to research on^118–120^. Micro-channel structures in thermal creep flow KPs have rectangular channels (Fig. 10a)^116,121–128^, curved-straight channels (Fig. 10b)^129–132^, double-curved channels (Fig. 10c)^133^, matrix channels (Fig. 10d)^94^, sinusoidal channels (Fig. 10e)^94^, and tapered channels (Fig. 10f, g)^134,135^, as shown in Fig. 10. Note that the flow mechanisms in all these structures are similar, i.e., thermal creep flow induced by the temperature gradients along the walls. For a detailed analyses on the generation of thermal creep flow, refer to the previous section “Thermal creep flow”.

The rectangular micro-channel is a two-dimensional abstraction of the traditional thermal creep flow KP structure, and has been thoroughly researched. Curved-straight alternated micro-channels have been put forward as the thermal creep effect in the curved section is stronger than that in the straight section^130,131^. The hydraulic diameter of this kind of channel is uniform everywhere, which is convenient for manufacturing in actual applications without requiring complex joints between sections. Likewise, in the double-curved and sinusoidal channels, steady flows are formed since the nonuniform variation of the hydraulic diameter of the channels breaks the nonequilibrium of the thermal creep effect. Although the construction of these two channel types also do not require complex joints between the sections, the complexity of the curved walls makes their manufacture more difficult. The matrix channel structure consists of four channels and nine uniformly distributed squares of the same size. This channel structure also faces the problem of complex fabrication. However, by artificially adjusting the temperature distribution of each square boundary, gases can flow in and out through any four channels, which cannot be achieved by other channel structures. By the way, among the five kinds of micro-channels discussed above (Fig. 10a–e), the rectangular and matrix channels have almost the same demonstrated performance and have the best performance, while the curved-straight channel has the worst performance^94^. Tapered (converging and diverging) micro-channels have highly similar structures to rectangular micro-channels, but the mass flow rate decreases significantly with increasing inclination angle. A diverging channel has a larger mass flow rate than a converging channel^134,136^. Therefore, in terms of fabrication, the curved-straight channel is probably the best choice for thermal creep KPs due to its simple structure and lack of requirements for complex joints between sections. Nevertheless, in terms of performance (mass flow rate and compression ratio), the rectangular and matrix channels perform better. In particular, the matrix channel has the ability to control the direction of the gas flow, an ability that the other channels do not have.

In summary, the micro-channel structure is one of the key factors that affect the gas flow and KP performance. The modification of existing structures and introduction of novel ones are always favored issues in KP research. The majority of the results from micro-channel studies have not yet been verified by experiments. The actual behavior of micro-channels needs to be experimentally confirmed to lay the foundations for actual applications.

### Thermal edge flow KP configuration

**Fig. 11:**
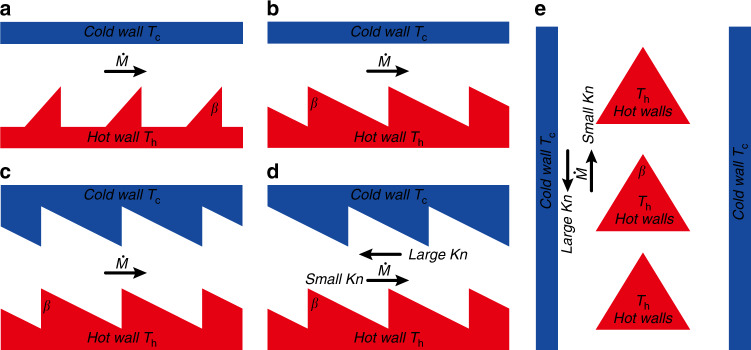
Thermal edge flow KP channel structures and configurations

Since thermal edge flow KPs were first put forward, several geometric shapes have already been proposed and employed in pumps^76^. Because thermal edge flow over the edges of a plate of zero thickness is an idealized situation^56^, studies of thermal edge flow KPs have mainly centered around ratchet-like structures. Figure 11 presents the micro-channel structures of thermal edge flow KPs, which are similar to some extent. It should be noted that in these channel configurations, the flow mechanisms are all similar, i.e., the thermal edge flows are induced by the sharp curves of the isothermal surface over the sharp edges. For the detailed analysis on this thermal flow, refer to the previous section “Thermal edge flow and radiometric flow”.

For the archetypical two-dimensional (2D) channel consisting of a cold flat wall and a hot ratchet surface shown in Fig. 11a, it is found that^80^ (i) only when the hot ratchet surface combines specular and diffusive segments (the inclined segment is specular while the horizontal and vertical segments are diffusive), can gas flow be observed; (ii) if all surfaces are diffusive, almost no gas flow is observed. However, later numerical analysis^77,137–142^ indicates that gas flow can be observed even when all the surfaces are diffusive. The tip angle plays an important role in the pumping effect. In order to induce a significant thermal edge flow, the angles *β* of the ratchet tips are all acute angles^77,80,137–142^. Thus, a ratchet surface composed of only an inclined section and a vertical section, as shown in Fig. 11b, was proposed. This kind of structure, however, presents added difficulties for manufacture. Putting aside the manufacturing difficulty and only considering performance (mass flow rate), walls composed entirely of ratchet structures (Fig. 11c, d) exhibit enhanced gas flow rates^137,139^. In this channel structure, the gas flow direction^140,142^ and pumping level^77,139,140,142^ can be easily controlled by adjusting the distance between two ratchets. Moreover, the net flow direction in the micro-channels can be reversed with the variation of *Kn*^140,142^. The two characteristics mentioned above are displayed in a ratchet structure configuration reported recently (Fig. 11e). Note that the energy efficiency of this channel configuration is better than that of other ratchet structures. The high energy efficiency results from the triangle hot plates being placed inside the two parallel cold walls. The hot plates are totally surrounded by gases and thus no heat is transferred to the external environment. In other structures, only one side of the hot wall (the upper side of the ratchet hot wall in Fig. 11a–d) is functional for the gas flow, while the other side has direct heat transfer with the external environment, and thus the energy efficiency decreases.

It should be also noted that compared with the channel structures of thermal creep flow KPs illustrated in the section “Thermal creep flow KP configurations”, the advantage of these kinds of KP structures is that their walls are isothermal instead of having nonuniform temperature distribution along the surfaces. Thus, the fabrication and temperature control of the thermal edge flow KP is much easier than that of the thermal creep flow KP, especially for more powerful KPs with multiple stages. For instance, a thermal creep flow KP requires a periodic temperature distribution with temperature gradients along the micro-channels and opposing temperature gradients along the connection channels. A thermal edge flow KP, in contrast, simply requires the addition of more isothermal ratchet surfaces without the need to address the problem of temperature control.

In fact, the authors of the works investigating the structures in Fig. 11^77,80,137,139–142^ have all discussed the influence of surface accommodation coefficients on the gas flow. When the accommodation coefficients of the adjacent walls are different, the thermalization of the gas molecules colliding with the walls is affected, and the gas flow is now driven by radiometric flow^56,57^. Radiometric flow is exploited in radiometric flow KPs, which will be discussed in the next chapter.

### Radiometric flow KP configuration

**Fig. 12:**
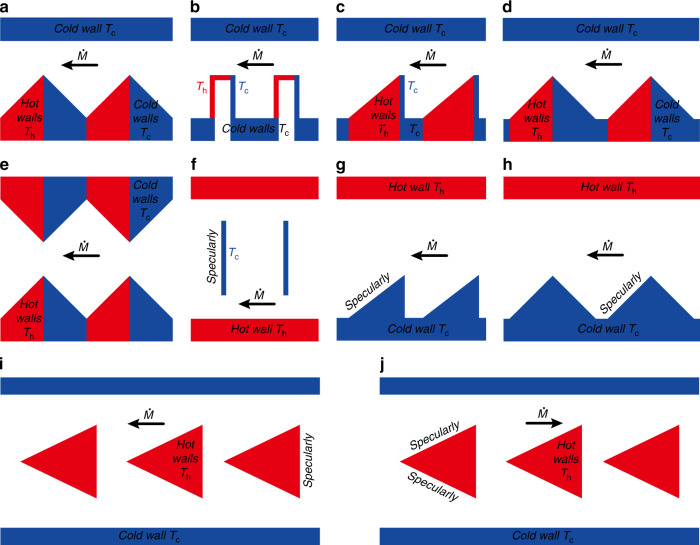
Radiometric flow KP channel structures and configurations

The configuration considered at the end of the last chapter is the basic structure of the radiometric flow KP^56,57^. The inspiration for the configuration comes from the Crookes radiometer^71,72,143^. There are fewer studies on radiometric flow KPs, which are still in the stage of theoretical analysis and numerical simulation. No relevant experiments have been reported yet. As mentioned above, the radiometric flow KP configuration can be realized by changing the temperature of the adjacent walls and accommodation coefficients. Some variants of radiometric flow KPs are shown in Fig. 12. Note that in all these channel structures, the flow mechanisms are similar, i.e., radiometric flow is induced by the nonuniform variation of the isothermal surfaces due to the different temperatures on the two sides of the walls (plates, vanes, and ratchets). The detailed analyses of the generation of radiometric flow are given in the previous section “Thermal edge flow and radiometric flow”.

Different temperatures are established for the walls, as presented in Fig. 12a–e. The temperatures can be set by using heaters, heat sinks or thermoelectric materials. Additionally, Fig. 12f–j shows the variants obtained by changing the surface accommodation coefficients. Although the reflections that occur on the surfaces are mostly diffusive with tangential momentum accommodation coefficients of around 1, accommodation coefficients of far less than 1 are present for certain special materials and gas−surface combinations^144–151^. This provides a reliable means to manufacture radiometric flow KPs through configuring the accommodation coefficients. Compared to the approach of setting up the wall temperature, configuring accommodation coefficients not only results in reduced KP volume, but also improved KP performance. The reason for these is that decreasing the accommodation coefficient will reduce the number of gas molecules, which exchange energy with the walls, especially for specular walls, with which no gas molecules exchange energy. Thus, specular walls do not require heat sinks. This hypothesis needs to be further researched on and experimentally verified.

Now, let us simply analyze the advantages and the disadvantages of the channel structures in Fig. 12. First of all, in terms of manufacturing difficulty, the fabrication of the configuration in Fig. 12e is the most complex while that of the configuration in Fig. 12f is the easiest. The dimensions of most of the channels are at the level of microns or nanometers. The walls of the configuration in Fig. 12e have the most ratchets, which are hard to manufacture while the configuration in Fig. 12f consists simply of plates, which can be manufactured easily. Therefore, the order of manufacturing difficulty of these configurations is Fig. 12e > Fig. 12d > Fig. 12h > Fig. 12a > Fig. 12c > Fig. 12i ≈ Fig. 12j > Fig. 12g > Fig. 12b > Fig. 12f. In terms of performance (mass flow rate), the structure shown in Fig. 12e has the best performance, while the structure in Fig. 12b has the worst performance. That is because gas flow is improved when the inclined segments are of low temperature (or specular) and the vertical segments are of high temperature (or diffusive)^82,140,142^. In addition, compared with the configuration having a single wall with the ratchet pattern, configurations where both the upper and the lower walls contain the ratchet patterns have a better gas flow^82,139^. Thus, the performances of these channel configurations can be sorted into the following order, Fig. 12e > Fig. 12j > Fig. 12i > Fig. 12a > Fig. 12d ≈ Fig. 12h > Fig. 12c ≈ Fig. 12g > Fig. 12f > Fig. 12b. Finally, let us consider the energy efficiency (or energy dissipation). The energy efficiency of the configurations in Fig. 12i–j is the highest while that in Fig. 12f is the lowest. The higher efficiency of the configurations in Fig. 12i–j is due to the placement of the triangle hot plates between the two parallel cold walls, so that there is no heat transfer with the external environment. In the other configurations, only one side of the hot wall is functional for the gas flow while the other side transfers heat directly with the external environment. Moreover, the configuration in Fig. 12f has two hot walls, which leads to more heat transfer with the external environment. Thus, the energy efficiency for these configurations can be ranked as follows: Fig. 12i ≈ Fig. 12j > Fig. 12b > Fig. 12c ≈ Fig. 12d > Fig. 12a > Fig. 12e > Fig. 12g ≈ Fig. 12h > Fig. 12f. Additionally, note that the specific quantitative analyses for the advantages and disadvantages of these configurations still require a large number of numerical simulations and experiments for comparison and validation.

In summary, it is difficult in practice to control the temperature gradient distribution along the walls, and the dimensions of the micro-channels are in the micron or nanometer scale, which makes fabrication complicated. Thus, thermal edge flow KPs and radiometric flow KPs composed of isothermal walls have better potential for applications than thermal creep flow KPs. The search for the optimal KP configuration is still an important point of research. Candidate configurations will need to be constructed and evaluated in the terms of manufacturing complexity, fabrication cost, performance (flow rate and compression ratio), and energy dissipation.

### Computational approaches

Experiments are the only way to verify theoretical work. However, limitations in current manufacturing and measurement technologies significantly restrict the development of experimental KP research. The emergence of high-performance computers and progress in numerical calculation methods have attracted much interest among scholars in using simulations to research on KPs. Numerical calculation plays an important part in investigating KP configurations and verifying the feasibility of each configuration. Although there are probably some differences between the simulation and actual experimental results, highly precise and diversified computational approaches have been developed.

The traditional Navier−Stokes (NS) equation is no longer applicable in the rarefied gas flow in KPs, and the Boltzmann equation should be used. However, gas flow can still be observed for small *Kn* when the NS equation is applied with appropriate velocity slip and temperature jump boundary conditions^119,132,139,152^. The results obtained from the Boltzmann equation are more stable and accurate, but require longer calculation times. Due to the complexity of the Boltzmann equation, scholars have applied different approaches to simplify the equation.

In order to solve the complex nonlinear collision integral, the linearized hypothesis was put forward and the linearized Boltzmann (LB) equation was obtained. With the help of the equation, the thermal creep flow problem can be solved and the KP performance evaluated^97,153–156^. However, the KP performance predicted by the equation is higher than that observed in the experiments^97^. The moment method makes some assumptions about the form of the distribution function, and obtains a number of equations by multiplying the Boltzmann equation with some molecular quantity and integrating over the entire velocity space^157,158^. The regularized 13-moment (R13) equations^159,160^ are the most often used equations in this method. However, R13 can successfully predict the mass and heat transfer of thermal creep flow with errors below 7% for the mass flow rate and below 10% for the heat flow rate^117,120^ only when *Kn* ≤ 0.5.

**Fig. 13:**
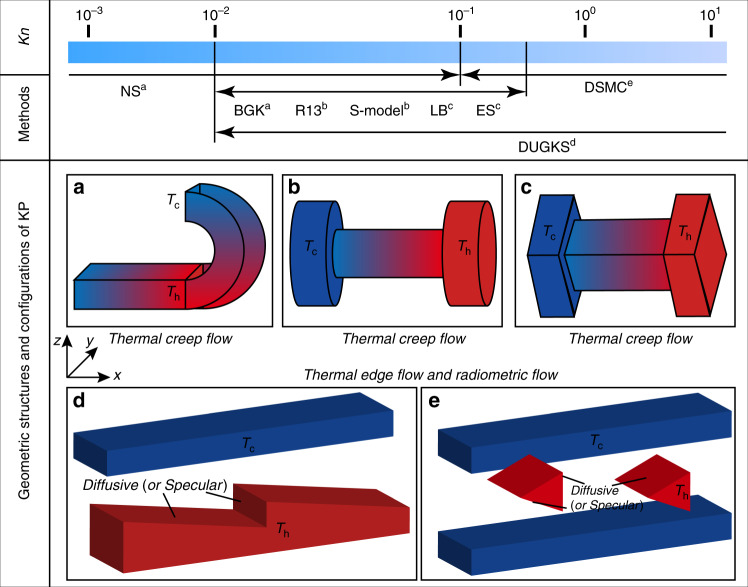
Computational approaches for KP numerical simulation research

The kinetic model equation is a plausible replacement for these methods. The equation replaces the complex collision term in the Boltzmann equation by a simplified collision term or collision model. The Bhatnagar−Gross−Krook (BGK) kinetic model is the most well-known model^161^. It uses a nonlinear relaxation term instead of the collision term in the Boltzmann equation. However, the Prandtl number in the BGK model is always 1, which does not accord with most actual scenarios. Therefore, the ellipsoidal statistical (ES) kinetic model^162^ in which the Prandtl number can take any arbitrary value was put forward. The Shakhov model (S-model)^163^ is also a common kinetic model. An accurate Prandtl number can be obtained by changing the heat flux in the model. Note that the models discussed above have good agreement for small *Kn*. Today, the discrete unified gas-kinetic scheme (DUGKS)^164–168^ based on the BGK and S-models is widely applied in KP research. Similar to other kinetic methods, its computational efficiency is also high for small *Kn*. In fact, these methods only solve equations which are similar to the Boltzmann equation. However, Direct Simulation Monte Carlo (DSMC)^169–171^ uses a large number of simulated particles to simulate the motion of real gas molecules in the flow field. The solution of the Boltzmann equation is obtained from the molecule statistics. The biggest drawback of the method is low computational efficiency, especially for a small *Kn*. However, the calculation results and experimental data have good agreement^172–175^. Figure 13 summarizes the numerical methods employed in KP research.

## KP performance research

For all types of KPs, a micro-pump system can be built out of serially connected (cascade or parallel) micro-channels to improve the performance. Models^87,122,129,156^ and methods^176–178^ that can effectively predict KP performance have already been reported. The indexes used to evaluate the performance of KPs are the pressure/compression ratio and the flow rate (flow speed), depending on the working mode of the KP. When the KP is used as a vacuum pump, attention should be paid to the pressure variation. When the pump is used to move fluids, focus should be placed on the flow rate. If the pressures at the two ends of the pump are equal, flow rate can reach the maximum. Moreover, efficiency measures the overall performance of the pump. Here, the research on KP performance is discussed in terms of the three aspects of pressure, flow rate, and efficiency.

### Pressure characteristics

At the end of last century, experimental analysis of the pressure parameter for a working KP was conducted^179,180^. Owing to the easy measurement of the pressure variation, the authors performed a large number of experiments on the KP pressure. However, the fabricated micro-channel dimensions were rather large, and the machined thermal creep flow KP^84^ and thermal edge flow KP^76^ were hence limited to functioning in a low-pressure environment. The compression ratio that the thermal creep flow KP provided was around 2 (ambient pressure of 10 or 20 Pa)^84^, while that of the thermal edge flow KP was about 1.4 (ambient pressure of 40 Pa)^76^.

Micromachining technology was later applied to the manufacturing of KPs, and the micro-channels were replaced by porous media. Under these circumstances, the pump can work at either low pressure or atmospheric pressure. However, different porous media affect the pressure characteristics of the pump to some extent. Among the four materials (porous glass (VYCOR), clinoptilolite, 5 bar porous ceramic, and 15 bar porous ceramic) discussed in the article^109^, 15 bar synthesized ceramic has the best performance.

**Fig. 14:**
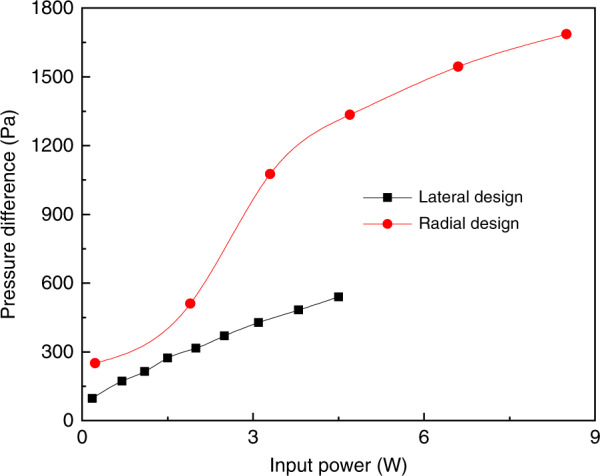
Comparison of pressure difference obtained as a function of the input power for the lateral design and the radial design^99^

The experimental results^101,102^ demonstrate that the smaller the pore diameter is, the higher is the pressure difference maintained. In addition, stacking multilayer membrane structures can provide better performance. Five layers of stacked membrane structures can provide about 2.3 times higher pressure difference than a single membrane structure^101^. However, for mixed cellulose esters with the same pore diameters and porosity, the maximum pressure differences reported by different articles^100–102^ have large differences. These differences are probably the result of factors such as gas species, membrane thickness, input power, and differing manufacturing techniques and structures of the pumping systems^181–183^. In fact, these factors affect KP pressure characteristics significantly. For example, although the radial and lateral pumps in an article^99^ have the same pore diameters, the pumps with a radial design have much higher performance than the pumps with a lateral design, as shown in Fig. 14. The pump with a lateral design has a maximum pressure difference of 539.9 Pa at an input power of 4.5 W, but when the input power is 3.3 W, the pressure difference of the pump with a radial design can reach 1076 Pa. In another example, using the same mixed cellulose esters with pore diameter of 100 nm, the maximum pressure difference can reach 63.4 Pa at the input power of 3.75 W through traditional heating using electrical resistance^102^, but the maximum pressure difference can reach 1686 Pa at the input power of 8.5 W through heating by thermoelectric modules^99^. This difference has aroused interest in the discovery of multifunctional materials. Recently, the use of a multifunctional nanometer porous thermoelectric material bismuth telluride (Bi_2_Te_3_)^112^ to manufacture KPs has been reported. The pores in the material are used as the gas flow channels while the thermoelectric property is used for creating the temperature difference for forming thermal creep flow. The pressure difference and mass flow rate increase significantly with the increase of the thermoelectric figure of merit^184^. The pressure difference can be increased further by decreasing the pore diameter. Such forthcoming changes can be anticipated.

**Fig. 15:**
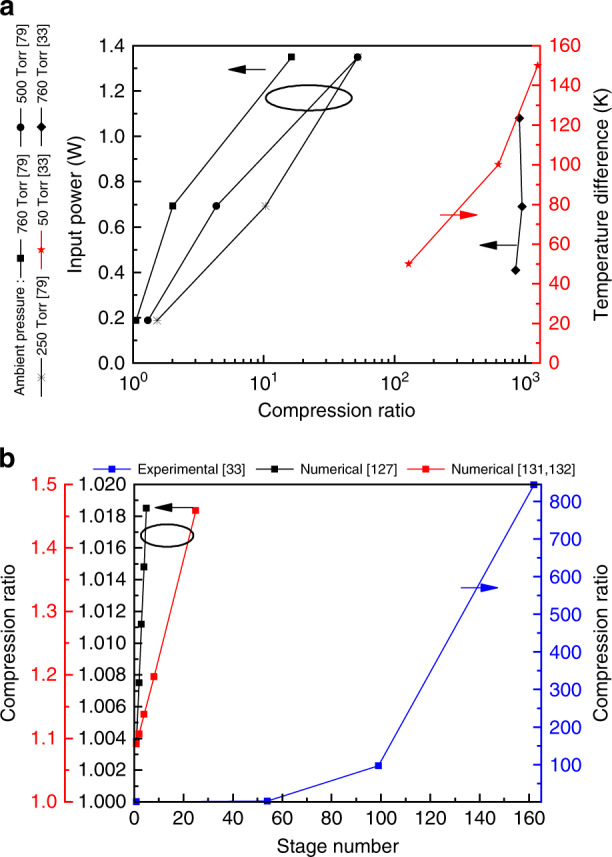
Compression ratios for different input energy and stage numbers. **a** The relation between energy and compression ratio. **b** The relation between stage number and compression ratio

With the progress in micromachining techniques, it is now possible to shrink the micro-channel dimensions. The first thermal creep flow KP manufactured by micromachining techniques was reported in 2005^90^. At the input power of 80 mW and atmospheric pressure, the compression ratio achieved by a single-stage KP is around 2.2^90^. According to Eq. (2), the most direct way to increase the compression ratio is to increase the temperature difference (input power) between the cold and hot ends. This method is, however, limited to the chosen materials used for manufacturing the KP. Cascading multiple stages can improve the pump compression ratio more effectively^33,79,89,91,185–187^. Figure 15 displays the relation of compression ratio to input energy and KP stage number quantitatively. Note that a KP with 162-cascading stages was reported in the articles^40,89^. The pump has two parts. The channel dimensions of the first 54 stages are small (narrow channels are 0.1 μm, wide channels are 30 μm). These stages decrease the pressure from atmospheric pressure to about 50 Torr. This use of a KP as a foreline pump reported in the articles^79,91^ can be regarded as an application of KPs. The channel dimensions of the last 108 stages are larger (narrow channels are 1 μm, wide channels are 100 μm). These stages are used in a low pressure environment to further decrease the pressure. The measured compression ratio of the pump reached up to 844 (760 → 0.9 Torr) at an input power of 0.39 W. If the input power is increased to 0.69 W, the compression ratio will reach 950 (760 → 0.8 Torr). This high compression ratio is not only the limit value of present KPs, but also beyond the capacity of any microroughing pump so far. However, the compression ratio does not increase significantly with the input power. At a higher input power of 1.08 W, the compression ratio will instead decrease^33^. The reason for the phenomenon may be due to ineffective heat dissipation of the equipment, resulting in a decrease in the temperature difference between the cold and hot ends. Here, it needs to be emphasized that effective thermal management contributes to improved pump performance^188^. For example, this situation will not appear if thermoelectric materials (thermoelectric modules) are used for heating^99,112^.

The channels for all the KPs discussed above can be regarded as rectangular structures. The compression ratios achieved with other forms of micro-channel configurations proposed recently^94,116,121–135^ have already been reported in the corresponding numerical studies. Table 1 records the maximum compression ratios of different varieties of KPs in simulation studies. From Table 1 and Fig. 15b, we can see that the influence of the micro-channel geometric structure on pump pressure properties is obvious. For a rectangular channel, the compression ratio increases linearly with the stage number, manifesting the single-stage replication characteristic^127^. The simulation results have some differences from the experimental results^33,79^, which display an overall nonlinear variation. In fact, the linear increase in compression ratio with stage number is only applicable to small stage number. However, enlarging the height or diameter of the connection channels can improve the compression ratio^127,189^. Additionally, in order to analyze the effect of surface roughness on the properties of KPs, micro-channel configurations with obstacles of different shapes (rectangle, triangle, semicircle) have been considered in the literature^190,191^. The simulation results demonstrate that increasing the widths of the rectangular obstacles contributes to increasing the pressure difference. For a triangle obstacle, this increase will appear only when the width of its base is large. In contrast, the pressure-rising property of the pump will be degraded if the obstacle height increases. Therefore, the surface roughness of the channel should be decreased to avoid decreasing the pressure-rising property.

**Table 1:** Compression ratios of thermal creep KPs with different geometric shapes and stage numbers

No. Sec.	1	2	3	4	5	8	16	25	Refs.
Fig. 10a	1.004	1.008	1.011	1.015	1.019	n*/*r	n*/*r	n*/*r	^127^
	n*/*r	n*/*r	n*/*r	1.253	n*/*r	1.309	n*/*r	n*/*r	^94^
Fig. 10b	1.091	1.107	n*/*r	1.138	n*/*r	1.197	n*/*r	1.459	^131,132^
	n*/*r	1.06	n*/*r	1.116	n*/*r	1.231	1.486	n*/*r	^130^
	n*/*r	n*/*r	n*/*r	1.077	n*/*r	1.081	n*/*r	n*/*r	^133^
Fig. 10c	n*/*r	n*/*r	n*/*r	1.088	n*/*r	1.118	n*/*r	n*/*r	^133^
Fig. 10d	1.313	n*/*r	n*/*r	n*/*r	n*/*r	n*/*r	n*/*r	n*/*r	^94^
Fig. 10e	n*/*r	n*/*r	n*/*r	1.166	n*/*r	1.294	n*/*r	n*/*r	^94^

### Flow rate characteristics

With the development of measurement techniques, it is now possible to measure the flow rate of some micro-devices, such as KPs. From the above discussion, it can be seen that the compression ratios of the majority of the pumps currently manufactured are not high. However, the flow rates of the pumps are highly reasonable, and the supplying of fluids can be completely achieved in a specific environment. A KP system manufactured by micromachining techniques reported in the literature^93^ can not only function in atmospheric environment, but also provide an air flow rate of more than 200 sccm, which is the highest rate since the beginning of KPs. In addition, as experiments have demonstrated^192,193^, some factors, such as gas species, rarefication degree of the gas (*Kn*), and temperature have strong influence on flow rate. For example, the mass flow rate of argon increases about ten times from the slip to the free-molecular regimes^192^. The mass flow rate of helium can increase by around 38 times when the transition regime with 50 K temperature difference changes into the slip regime with 70 K temperature difference^193^. Channels with larger dimensions have better gas flow and increased pump flow rate intensity^187,192,193^. This demonstrates that under a given pressure, changing the channel dimension to adjust the rarefication degree of the gas can both satisfy the KP working conditions as well as improve the properties of the pump. Note that increasing the channel width can increase mass flow rate to some degree, but the mass flow rate will tend to saturate instead of increasing infinitely^187^.

**Fig. 16:**
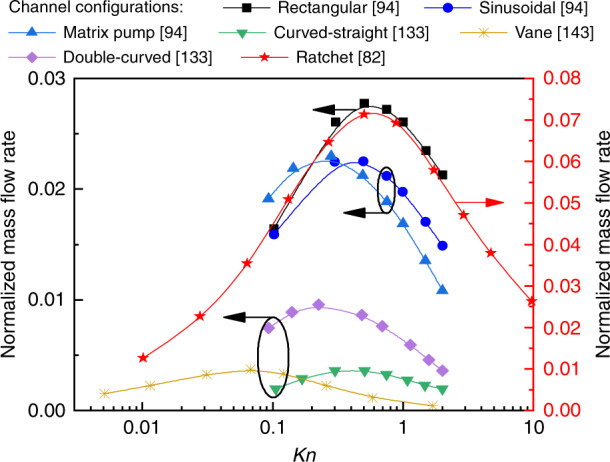
Relation of *Kn* to mass flow rate

The majority of the modern numerical analysis works use the (nondimensional) mass flow rate to describe the flow rate. Here, the influence of different pressures (*Kn*), temperatures, surface accommodation coefficients, geometric structures and dimensions on flow rate are considered. Table 2 presents the maximum flow rates of the different geometric structures reported in the past few years. We can see that flow rate is highly sensitive to the geometric structure. Rectangular layouts have the highest performance while the performance of the curved-straight geometric structure is the worst. For the tapered channels, the diverging parts have a higher flow rate than the converging parts. Divergence angles play important roles in improving mass flow rate. The mass flow rate always decreases with the increase of channel inclination^134^. Note that this conclusion contradicts later research^135^, because the connection channels of the models are not considered in the latter. In fact, the connection channels influence the flow field patterns and mass flow rate in micro-channels^194^. This is because the addition of connection channels will affect the inlet and outlet conditions at the two ends of micro-channels. In addition, increasing the aspect ratio can also increase the mass flow rate^135^. In particular, for a large *Kn*, the aspect ratio has more influence on the mass flow rate than the divergence angle^135^. Therefore, it may be said that if the gases are in the free-molecular limit (*Kn* ~ 50), the maximum flow rate will be attained. As a matter of fact, the maximum mass flow rate of the pump is usually obtained for an intermediate *Kn* (transition regime)^77,82,135,140,142^, as shown in Fig. 16. That is because with the increase of *Kn*, the number of gas molecules decreases and no gas flows at the two limits (*Kn* = 0 and *Kn* → ∞).

**Table 2:** Maximum flow rates and corresponding *Kn* for different geometries of KPs in numerical studies

Geometry	Nondimensional mass flow rates (10^–3^)	*Kn*	Refs.
Fig. 10a	28.0	0.53	^94^
Fig. 10b	3.7	0.38	^133^
Fig. 10c	9.6	0.24	^133^
Fig. 10d	23.0	0.24	^94^
Fig. 10e	23.0	0.39	^94^
Fig. 11d	1.81	0.09	^140^
Fig. 11e	2.83	0.15	^142^
Fig. 12b	20.24	0.51	^82^
Fig. 12e	71.08	0.61	^82^
Fig. 12f	3.69	0.07	^143^
Fig. 12g	7.54 (Volume flow rate)	0.1	^77^
Fig. 12i	42.2	0.11	^142^
Fig. 12j	86.8	0.11	^142^

**Fig. 17:**
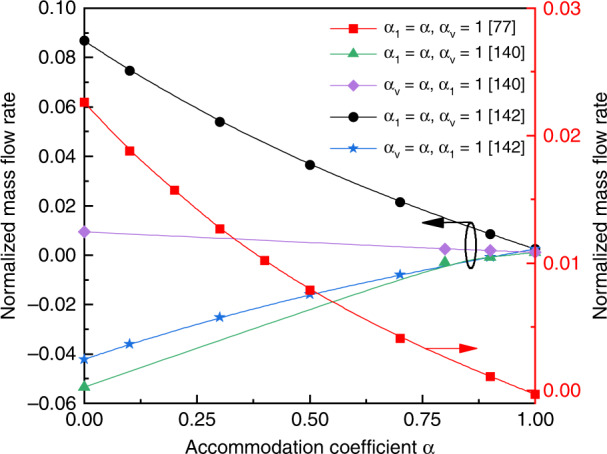
Relation of accommodation coefficient to mass flow rate

As discussed above, the surface accommodation coefficient has significant influence on the gas flow. In fact, it also triggers the variation of mass flow rate. Figure 17 displays the simulation results for the change of mass flow rate with the surface accommodation coefficients in different geometric structures^77,140,142^. Several conclusions can be drawn: (i) the mass flow rate varies significantly with the accommodation coefficient, (ii) the mass flow rate increases with the decrease of accommodation coefficient, and (iii) when the inclined walls are cold (specular), radiometric flow will be more prominent. The change rules (linear or nonlinear) differ between different structures. Please note that these results are obtained based on Maxwell gas−solid interaction models. However, the CLL (Cercignani−Lampise−Lord) model is considered to be better for modeling the interaction between the gas and surface^195,196^ because the model contains two independent accommodation coefficients, the normal thermal accommodation coefficient (NTAC) and tangential momentum accommodation coefficient (TMAC). Research^197^ has found that the mass flow rate is much more dependent on TMAC than on NTAC. The mass flow rate increases dramatically with the decrease of TMAC in the free-molecular and transition regimes, whereas in the slip regime, the mass flow rate tends to decrease slightly with the decrease of TMAC, because effective gas flow cannot occur in the slip regime.

**Fig. 18:**
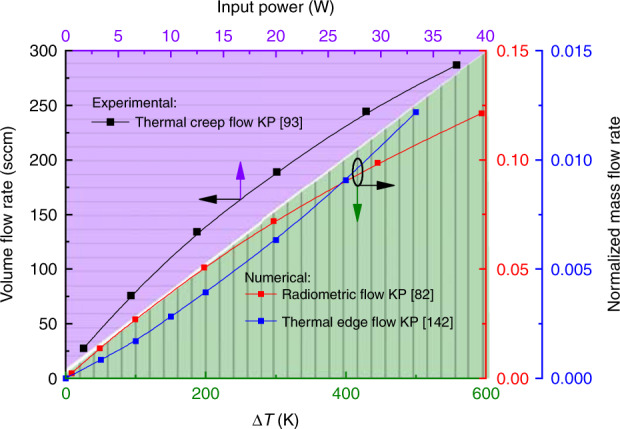
Flow rates for different temperature differences and input powers

The effects on the mass flow rates discussed above are all based on the assumption of constant temperature. Increasing the temperature difference (input power) will certainly increase the mass flow rate^99,130,192,193^, as presented in Fig. 18. Moreover, the temperature distribution along the micro-channel walls (linear temperature variation, step-wise temperature variation, nonmonotonic distribution) obviously affects the mass flow rate^114,116^. As expected, a linear temperature variation gives the highest mass flow rate. Compared to the mass flow rate for linear temperature variation, the mass flow rate for step-wise temperature variation is reduced by about 3%. The temperature peak value of the nonmonotonic distribution occurs between the two ends of the channel, resulting in reverse thermal creep flow, which causes the mass flow rate to decrease by about 18%. This fully demonstrates that temperature difference of the walls is the mechanism behind the thermal creep flow KP, but also limits its performance.

The thermal edge flow KP and radiometric flow KP consist of constant temperature surfaces to induce gas flow. Establishing a temperature difference between the cold and hot walls is a key factor for improved mass flow rate. Early research^139,140^ has assumed that the variation of mass flow rate with temperature difference is linear. Note that recent research^82,142^ has found this assumption to be invalid. In fact, the mass flow rate increases nonlinearly with temperature difference; the linear variation only occurs for a small temperature difference (*Kn*), as shown in Fig. 18. Obviously, the relations of the flow rate to the temperature difference (or the input power) of these three thermally induced flows (thermal creep flow, thermal edge flow and radiometric flow) are similar. That is, they all display nonlinear variations, but some differences exist in the data. These differences exist because in the figure, the relation between the volume flow rate to the input power is depicted for the thermal creep flow KP, whereas the relation between the nondimensional mass flow rate and the temperature gradient is depicted for the thermal edge flow KP and the radiometric flow KP. Differences may also result from the different configurations and parameters of these three KPs in aspects such as geometric structure, geometric dimension, gas species, and gas pressure. Note that the linear relation can be used to judge the applicability of the KP in MEMS to some extent as, in general, the working temperature difference of MEMS cannot be larger than 200 K.

### Relation between pressure and flow rate

In order to discuss the performance of KPs further, the relations between pressure and flow rate in KPs with different working patterns are analyzed. It is well-known that there are two kinds of flows in the micro-channels of thermal creep flow KPs, Poiseuille flow and thermal creep flow. For a single-stage KP with two connected cavities, thermal creep flow is formed by the temperature difference between the cavities. As timeΔ*T* ≤ 200 K goes by, a pressure difference *t* appears between the two cavities, resulting in Poiseuille flow, which hinders the thermal creep flow. The relation between mass flow rate per unit area Δ*P* and pressure difference *Ṁ* can be represented by^153,178^

3 $$M = \frac{V}{{2{{RT}}_rA}}\frac{{{\mathrm d}({\mathrm{\Delta }}P)}}{{{\mathrm d}t}}$$

where *V* is the volume of the cavity, *R* is the gas constant, *T*_*r*_ is the room temperature, and *A* is the cross-sectional area of the micro-channel. Δ*P* is a function of time *t*^192,198^,

4 $$\Delta P(t) = \Delta P_{\max }(1 - e^{ - t/\tau })$$

where Δ*P*_max_ is the pressure difference at steady state and *τ* is the characteristic time constant. Through the removal of *τ*, the final relation between pressure difference to mass flow rate can be obtained,

5 $$\dot M(\Delta P) = \frac{V}{{2\tau {{RT}}_rA}}(\Delta P_{\max } - \Delta P)$$

**Fig. 19:**
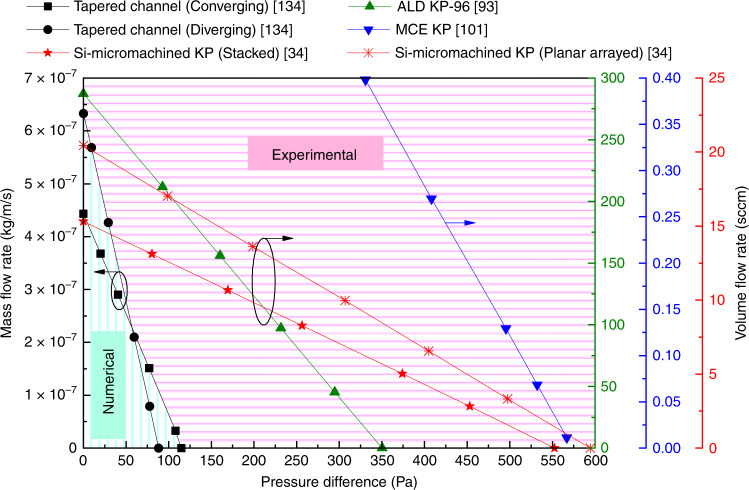
Flow rate versus pressure difference

As expected, the maximum mass flow rate appears when the KP begins to work, or in an open system, i.e., at *t* = 0 or Δ*P* = 0. Moreover, the mass flow rate decreases linearly with the increase of pressure difference, which can be seen from the numerical^134,199^ and experimental^34,93,100^ results shown in Fig. 19. It should be noted that the influence of pressure difference on the mass flow rate is considered in the numerical simulations, but the variation of volume flow rate with the change of pressure difference is measured in the experiments. Thus, differences exist between the simulation and experimental results. Additional differences in the results may also result from the differences in geometric structures, geometric dimension, gas species, temperature difference, and rarefaction parameter (Δ*P* = 0). In fact, this qualitative conclusion is also applicable to multistage thermal creep flow KPs to some degree. Additionally, shrinking the micro-channel length will decrease the compression ratio to a certain degree, but will also improve the mass flow rate^189^. Note that Eq. (5) is obtained based on the thermal creep flow KP. There are still no mathematical expressions for the mass flow rate and pressure difference applicable to the thermal edge flow KP and the radiometric KP, but the variation trends of mass flow rate with pressure difference are similar in all three types of KPs.

### Efficiency characteristics

In order to evaluate the integrated performance of KPs, it is necessary to consider their efficiency characteristics. The efficiency of a KP can be formulated in a way similar to that of a macro-scale pump, that is, as the ratio of the output power (useful work) to the heat input^200–202^. Thus, for a thermal creep flow KP, the formula of the ideal pumping efficiency neglecting thermal loss (the output power is the flow work performed by the pumping gas of the KP) can be written as^200–202^

6 $$\eta _{{\mathrm {ideal}}} = \frac{R}{{C_p}}\frac{{\sqrt {T_{\mathrm h}/T_{\mathrm c}} - P_{\mathrm h}/P_{\mathrm c}}}{{P_{\mathrm h}/P_{\mathrm c} + \sqrt {T_{\mathrm h}/T_{\mathrm c}} }}$$

where *C*_*p*_ is specific heat, and *T*_c_*, P*_c_ and *T*_h_, *P*_h_ are the temperature and pressure of the cold and hot cavities, respectively. Evidently, the ideal pumping efficiency increases nonlinearly with the temperature ratio (or temperature difference), and the maximum efficiency tends to be the ratio of the gas constant *R* to the gas-specific heat *C*_*p*_*.*

In order to calculate the pump efficiency more realistically, thermal losses cannot be neglected. However, once the thermal loss is considered, the efficiency of the pump decreases dramatically^201,202^, which can be expected. Note that the length of the channel is irrelevant to the efficiency, but the shape of the cross-section will affect the efficiency including thermal losses because the cross-section shape affects the transmission probability of the channels. Generally, increasing the hydraulic diameters of the micro-channels improves the efficiency of the pump^194^.

On the other hand, when the KP is used as a vacuum pump and at steady state, the efficiency of pump is zero from Eq. (6). That is because the above efficiency equation only considers the KP flow rate characteristics, but does not consider its pressure characteristics^189^. To be more precise, increasing the pressure difference between the cold and hot cavities reduces the flow work done by the pumping gas of KP (net flow rate), and thus the pumping efficiency decreases. In the state of equilibrium, the net flow rate through a KP is zero, i.e. the pumping efficiency is zero. This means that the pumping efficiency only considers the flow rate characteristics of the gases that move from the cold (low pressure) cavity to the hot (high pressure) cavity, but excludes the pressure-raising property of the KP. Therefore, in order to assess the efficiency property of KPs more accurately, other several definitions for KP efficiency were proposed by Klein^189^. These efficiency equations were obtained only by numerical calculation, and have not been verified by experiments.

Please note that this efficiency is not necessarily applicable to thermal edge flow KPs and radiometric flow KPs. Issues with radiometric flow KPs have been discussed in simulation works for the past few years^82,141,203^. The radiometric flow KP efficiency is considered to be the ratio of output power *P*_*w*_ to heat flux of the walls *q*^82^:

7 $$\eta = \frac{{P_w}}{q} = \frac{{F_xU_{x,{\mathrm {avg}}}}}{q}$$

where *F*_*x*_ is the force density on the walls and *U*_*x*,avg_ is the average velocity. The efficiency of the pump reaches the highest value in the transition regime, but the value is small and is less than 1%^82,141^. Moreover, the influence of the channel geometric parameters and surface accommodation coefficients on the efficiency is significant^141,203^.

In summary, research on KP efficiency is still scarce and immature, and the relevant theories still have a lot of room for improvement. More numerical and experimental research need to be performed to determine the actual rules for improving KP efficiency.

## Research on KP applications

### Vacuum generation

The most basic application of KP is in micro-pumps. In fact, generating vacuum is one application of the KP pressure-rising property discussed in the section “Pressure characteristics”. A KP system directly manufactured by the machining of wide-narrow circular glass pipes can reduce the pressure of an 8000 cm^3^ reservoir by 50% within 300 s (Δ*T* = 150 K, and *P*_out_ is 10 or 20 Pa)^84^.

Aerogel membranes were used for the first partially micro-machined thermal creep flow KPs. Using helium as the working medium, the optimal compression ratio of the pump at atmospheric pressure is about 1.02^95,180^. A few years later, aerogel membranes were applied to fabricate a 15-stage KP system. The system can maintain a pressure difference of 15.8 kPa^96^ when illuminated by a radiant flux of 20.9 mW/cm^2^. A KP using aerogel membranes and functioning in a low pressure environment^97^ can produce 150 mbar pressure difference with 15 cascading stages. Recently, a novel and simple sol−gel process was reported and applied to fabricate aerogel membranes^98^. The KP manufactured with these aerogel membranes can function at a temperature of 400 °C, which contributes to an improved compression ratio. However, in that paper, only the flow rate, and not the pressure information, was reported.

Moreover, a KP fabricated with natural zeolite can maintain 2.5 kPa pressure difference with a single stage^106,107^. Through multistage extension, the pressure difference is expected to reach 50 kPa (not reported in the paper)^106^. In references^108,109,204^, using multistage cascades, KP systems using 15 bar synthesized ceramic can maximally provide more than 12 kPa pressure difference. In a KP fabricated with mixed cellulose ester, the pressure difference can reach up to 1.05 kPa^101^ through the stacking of multilayer membrane structures. However, the maximum pressure reported in another article^102^ is only 157 Pa. The reasons for this are mentioned in the analysis in the section “Pressure characteristics”. In addition, employing thermoelectric materials as the heater/heat sink of KPs can increase the temperature difference between two cavities, improving the compression ratio^99^. Multifunctional thermoelectric materials have also been used to fabricate a KP^112^ that generates a maximum pressure difference of 300 Pa with an input power of 3.32 W.

The first single-stage KP completely fabricated by micromachining technology was based on Si materials^90^. The KP can lower the pressure in the 8 × 10^4^ μm^3^ cavity to 0.46 atm within only 2 s. However, the system cannot be applied to systems with larger volumes. Later, through the application of etching/lithography techniques and cascading stages, the KP compression ratio improved substantially. For example, a 48-cascading stage thermal creep flow KP fabricated by an etching technique was reported in 2012^79,91^. When the pump functions at atmospheric pressure with an input power of 1.35 W, the compression ratio is 15 (760 → 50 Torr). Using the same input power, when the pump is in a low-pressure environment (250 Torr), the compression ratio of the pump can reach up to 50 (250 → 5 Torr). In order to attain a larger compression ratio, the same research group proposed a 162-cascading stage KP^33,89^. The maximum compression ratio of the pump reaches up to 950 (760 → 0.8 Torr). Compared with the KPs reported in the past, such a high compression ratio is unprecedented. A few years later, this research group reported several kinds of micro-machined KP systems as well^34,93^. However, this time, the KPs can only provide around 1.03 compression ratio. This small value seems to not have interested any scholars. The pump can, however, provide an enormous flow rate (>200 sccm). Recently, a KP fabricated by silicon deep RIE (reactive ion etching), thermal oxidation, and anodic bonding processes^205^, which can function at atmospheric pressure was put forward. The pump is capable of decreasing the cavity pressure to about 10 kPa (compression ratio is around 10) at a temperature difference of 25 °C.

Please note that the above descriptions report the applications of thermal creep flow KPs for generating vacuum. Reports on the applications of thermal edge flow KPs and radiometric flow KPs systems are rare. The first thermal edge flow KP system can only function in a low pressure environment, and the maximum compression ratio is about 1.4 (40 → 28.8 Pa)^76^.

### Fluid delivery

Since a KP has flow rate characteristics, it can serve as a power/pumping system of an MEMS/NEMS device to deliver flow for the whole system. The first equipment to use a KP for flow delivery is a self-sustaining, self-pressurizing combustor^206–210^. This combustor, when combined with a SOFC (solid oxide fuel cell), can be used as a miniature power generator. A KP was first applied in 2011 in a micro-gas chromatography (μGC) system^211^. The experimental results demonstrate that when the carrier gases are N_2_ and dry air, the KP can provide a gas flow of 1 mL/min and 0.26 mL/min to the system, respectively. In order to shrink the dimensions of the μGC system, the crucial components (KP, preconcentrator, separation column, gas detectors) are all fabricated by lithography^212,213^. The μGC components can be integrated into a system by direct stacking without using capillaries for connections. This improves the performance of the system. The authors later used a bidirectional manipulation KP in the μGC, improving the analysis performance of the system further^214–217^. KPs can also be found in the micro systems of gas sensors^218^ in which the KPs pump the tested gases in the systems. KPs have also been used to drive gas flows in optomechanical devices for ultra-sensitive measurements^219^. The forces from the gases act on and deflect the microspikes in the devices. The deflections reflect the variation of the resonator resonant frequencies with the quantities measured.

A KP serving as an infusion pump in which the body temperature provides the heat source for the KP to pump low flow medicine for skin wounds was reported in 2013 ^220,221^. Please note that the KP does not pump gases, but generates a pressure difference to deliver medicine. Similarly, an integrated KP is used to pump a liquid through microfluidic channels on microscopic glass slides.

Recently, a new refrigeration system comprising a KP and vortex tube was reported^222^. The energy for the KP comes from low-grade residual/waste heat. The energy consumption is decreased substantially compared to traditional refrigeration systems. Moreover, KPs have also been used to design a new low energy consumption heat pump system^223–226^. Using a single-stage KP, the demonstrated output power of the system is 3.09 W^226^. A system containing a 30-stage KP produces an output power of 1.27 kW (theoretical prediction) at a temperature difference of 6 K^225^. Compared to energy conservation and consumption reduction, the development of renewable energy sources seems to have attracted more attention. A method for gathering solar energy based on the thermal creep effect has been put forward^227,228^, in which solar energy is converted to gas flow kinetic energy using a KP. The simulation results show that the energy conversion efficiency is 7.4%. Optimizing the system structure and working parameters will probably improve the conversion efficiency. Note that this method is also applicable to other renewable energy sources and low-grade residual/waste heat.

The application of thermal edge flow KP in flow delivery is much more limited. In early constructs, a thermal edge flow KP was applied in the freeze-drying process^229^. The experiments demonstrate that the KP facilitates efficient freezing and drying, shortening the drying time by 50%. Lately, a heat engine model based on thermal edge flow and radiometric flow KPs was proposed^57,141,203^. The gas flow in the channels and the tangential force acting on the walls are the keys to the functioning of the heat engine, which provides a new mechanism for converting thermal energy to mechanical energy. However, the conversion efficiency is greatly influenced by the configuration of the geometric structure. More research, in particular experimental analysis, is required to determine the actual behavior of the heat engine.

### Gas separation

The thermal creep effect in different gases differ from one another as gases with lighter molecules flow faster^103,230–232^. Thus, KPs can be used for gas separation. This is a unique characteristic of KPs absent in other pumps. In 2007, a mathematical model in which a KP is used as a gas separation device was first proposed^230^, verifying the feasibility of KPs for gas separation. Research has also found that increasing the cascading stage number of KPs improves the degree of separation, but lowers the time efficiency^186^. Thus, the degree of separation and time efficiency should be considered comprehensively. Later literature^233^ reported an open system gas separator simulated and studied using the DSMC method. The gas separation device comprises micro-channel arrays based on the thermal creep effect. The authors later simulated a system in which the main channel is replaced by a U-bend channel^234,235^. They found that the gas separation system with a U-bend channel gives a larger variation of the mole fraction, making the gas separation more effective.

In recent years, KPs using porous membranes (MCE membranes) as micro-channels have also been applied in gas separation devices^103–105,236^. One such gas separation device also uses a U-bend channel^103,104^. Up to 15% variation of mole fraction is observed when the average volume flux Q is 100 sccm. Another gas separation system is composed of two KPs^105,236^. One KP functions at a relatively large temperature difference (Δ*T* = 100 K), serving as a pumping element for the whole system to deliver mixed gas. The other KP operates at a relatively small temperature difference (Δ*T* ≈ 20 K), and is used to perform gas separation by inducing molecular exchange via the membranes. When supplying the gas volume flow rate of 5 sccm, the mole fraction difference between the two product gases is 5.5%, which can be expected. Recent research^237^ demonstrates that increasing the width of the main channel, decreasing flow speed, and enlarging the membrane length/area improve the gas separation performance of the devices. Moreover, the gas separation capability of KPs is also employed in gas sensors^238^. In these sensors, the KP generates different concentrations of different gas species in different places, leading to the variation of resonator resonant frequency, which can be used to indicate the gas species and concentration.

Besides the thermal creep flow KPs used for gas separation, thermal edge flow KPs also have the capacity to separate gases. As demonstrated in experiments^239^, the concentration of weightier gas molecules is higher in the high-pressure side while the concentration of the lighter gas molecules is higher in the low pressure side. However, reports on this phenomenon are rare. This is probably because the gas separation performance of thermal edge flow KPs is relatively weak. Recently, a gas separation device consisting of stretched filaments at different temperatures was studied^240,241^. The simulation results demonstrate that the gas separation effect is significant. The gas flow and separation are due to the thermal edge flow around the stretched filaments. The development and design of gas separation devices based on thermal edge flow KPs and radiometric flow KPs are thus of value and significance.

## Discussions and outlook

**Fig. 20:**
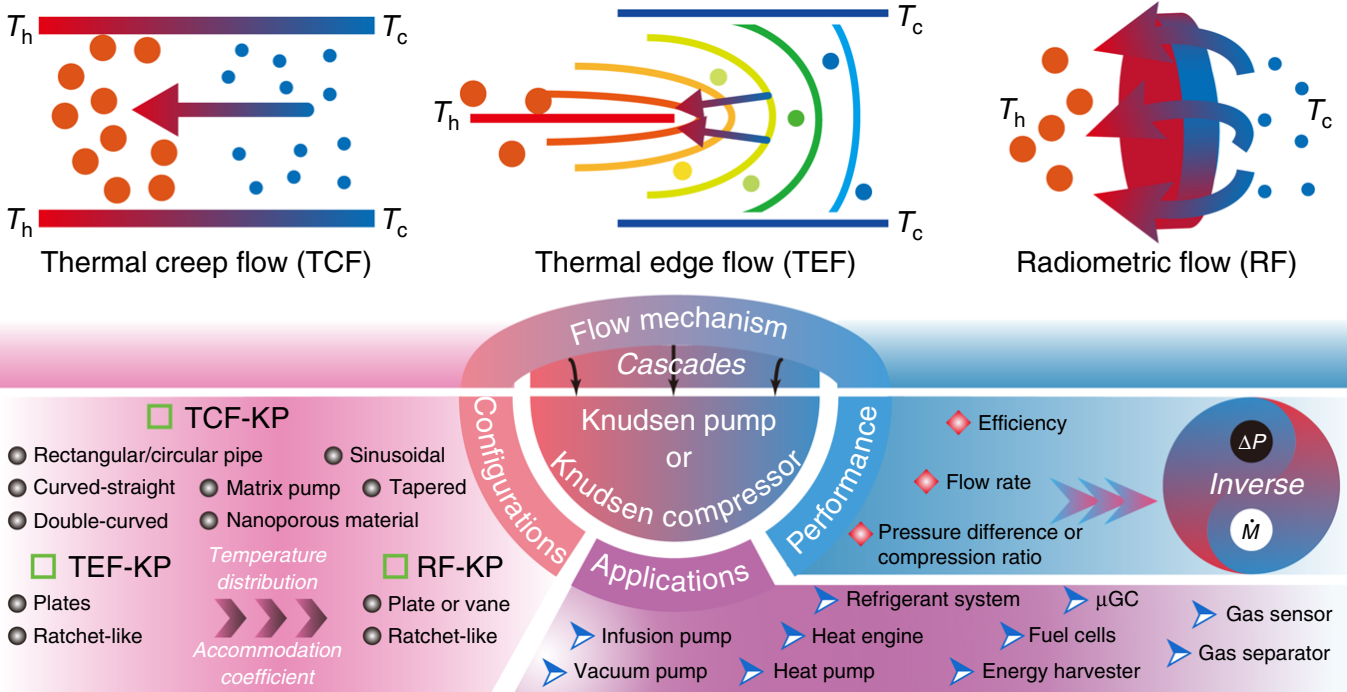
A simple schematic summary for KP research status at present

In this section, the future directions for KP development are outlined based on the past KP research. A brief summary of KP research is presented in Fig. 20.

### Exploring new KPs

Micro-channels are the key elements in KPs that ensure that gases are in a rarefied state. A steady flow of rarefied gas is achieved by temperature fields. Micro-channels and temperature fields directly determine the structural shape and performance of KPs. Since the emergence of KPs in 1909^38^, various variants of KPs have been put forward. The basic geometric structures and mechanisms for the gas flow in different KP variants are listed in Fig. 20. Based on the above conclusions and illustrations, the following key comments can be made:

1. Many kinds of simple geometric structures have already been proposed^94,121–135^ for early thermal creep flow KPs, as shown in Fig. 10. In contrast, the geometric structures of thermal edge flow KPs and radiometric flow KPs are mainly plates and ratchets (e.g. Figs. 11 and 12)^82,137–143^. More research is needed to explore the applicability of new channel geometries in KPs. Channel structures are not limited to 2D geometric shapes, and channels with 3D geometric shapes such as cylinders, prisms, circular cones, and pyramids are expected to be developed.
2. Channel configurations with only a single geometric shape might not satisfy the work requirements of high-performance KPs (high flow rate, large compression ratio)^94^. It is predicted that the future channel configurations of KPs will combine several geometric shapes. For example, in a channel composed of two different geometric shapes, like rectangles and ratchets, the gas flow will be controlled by both thermal creep flow and thermal edge flow (or radiometric flow). This construction might provide a larger flow rate and compression ratio, and realize the control of gas flow direction.
3. A new method for designing KPs is to use the pores in porous media as micro-channels^95–112^. In the future, porous materials having the characteristics of high microporosity, low thermal conductivity and low cost, like the nanoporous thermoelectric material Bi_2_Te_3_ proposed by Faiz et al.^112^, are expected to be applied in KPs. This will require much preparation work and multidisciplinary knowledge (e.g. material science, physics, chemistry, manufacturing, and mechanical engineering).
4. Changing the surface accommodation coefficients and temperature configurations on different walls can be used to control the flow mechanisms of thermally induced flows and realize KPs with different thermally induced flows^77,82,140,142,143^. For instance, the thermal edge flow KP is converted into a radiometric flow KP in the above discussion. This is important for the design, optimal performance, and applications of KPs, as well as for the exploration of new types of KPs.

### Improving KP performance

KP performance is mainly reflected in the compression ratio, flow rate, and efficiency, presented in Fig. 19. The compression ratio and flow rate are usually in an inverse relationship (linear negative correlation) in which they will never reach the optimal state simultaneously. Depending on the intended usage (vacuum generation and fluid delivery), the indexes for measuring KP performance are also different. Efficiency issues are, however, common to all applications. Based on the above conclusions and illustrations, the following key comments can be made:

1. The maximum compression ratio for reported KPs at present reaches up to 844 ^33^, but the flow rate is small. On the contrary, for KPs having small compression ratios, the maximum flow rate is more than 200 sccm^93^. With the appearance of micromachining techniques and multifunctional materials, improving the compression ratio and flow rate of the KP will be key focuses for research in the future.
2. The maximum pumping velocity of the KP used for generating vacuum is not high and also gradually decreases with the increase of compression ratio (e.g. Fig. 19)^33,93^. In order to shorten the pump-down time, we can increase the micro-channel dimension appropriately to improve the pumping velocity, but this will decrease the compression ratio. Thus, the integrative performance (compression ratio and flow rate) needs to be assessed when constructing KPs. For example, multichannel parallel connections can be applied to improve the pumping velocity without impacting the compression ratio of the KP itself^125^.
3. Efficiency is one index to assess the integrative performance of KPs. However, theoretical research on efficiency models is scarce and not comprehensive. There is still much room for improvement^82,141,200–203^. In current experimental and numerical simulation works, efficiency is rarely analyzed. More attention should be paid to efficiency in future research. One area of concern is the low energy efficiency in energy gathering and conversion^57,141,203,227,228^. In the future, the system structure can be optimized, system materials improved, and the heat dissipation of KPs decreased to improve the energy efficiency.

### Expanding the applications of KPs

To some extent, the applications of KPs drive themselves to progress and development. Based on the different properties of the applications, the applications can be classified into three main categories: (i) vacuum generation, (ii) flow delivery, and (iii) gas separation. The recent applications of KPs are listed in Fig. 20. Based on the above conclusions and illustrations, the following key comments can be made:

1. Since KPs were first proposed by Knudsen^38,39^, they have developed significantly as vacuum pumps for generating vacuum. Present Knudsen vacuum pumps can maximally decrease the pressure of cavities from atmospheric pressure to about 0.8 Torr^33^, which is the limit value of the microroughing pump. Through collocation with medium and high vacuum ion-sorption micro-pumps^242,243^, the pressure can be decreased further. It is predicted that the development of high compression ratio KPs will continue in the future. In addition, techniques for lowering pressure with KPs are expected to be applied in some high-pressure sampling devices. For example, adding KPs in devices like mass spectrometers can ensure that testing can performed in atmospheric environment.
2. KPs used for flow delivery have already been widely employed in micro-gas chromatographs^211–217^. In recent years, they have also been used in medicine delivery^220,221^ and in energy gathering and conversion devices^226–228^. Without doubt, KPs are expected to play important roles in bioengineering, microchemistry, medical diagnosis and analysis, and power engines in the future.
3. KPs have been used for gas separation since 2007. Gas separation is achieved with thermally induced gas flows^230^. Previous research has demonstrated that the channel dimension, KP size, and gas flow speed affect the degree and time efficiency of gas separation^186,232^. Gas separation in KPs is also utilized in a new method for testing gases^238^. All these imply that KPs would also be applied to gas recycling and removal in the future.

## Conclusions

According to the review above, the following conclusions can be drawn:

1. With further study, the application of KPs in MEMS/NEMS will increase, since KPs offer the advantages of having no moving parts, simple structures, easy construction, easy expansion, wide energy sources, and low energy consumption.
2. KPs can be classified into thermal creep flow KPs, thermal edge flow KPs, and radiometric flow KPs based on their thermal flow mechanisms. Thermal creep flow is induced by the temperature gradient along the walls. Thermal edge flow is triggered by dramatic changes in the temperature fields in the vicinity of the constant temperature edges. If the adjacent edges have different temperatures, radiometric flow will appear. In the future, new KPs based on other thermally induced flows may emerge.
3. Micro-channels are the key elements of KPs. The micro-channel structures not only influence the form of the gas flow fields, but are also directly related to the KP performance. Micro-channel structures can be obtained by micromachining techniques, and by using natural and artificially synthesized porous materials. In addition, surface accommodation coefficients and temperature configurations modulate and control the flow mechanism of the pump. In terms of the channel structure, apart from single 2D geometric shapes, the channels might also use 3D geometric shapes or combine many kinds of geometric shapes. In summary, the construction and relevant configurations of new channels are crucial in improving the performance of KPs, which is one of the goals for future research.
4. Research on KP performance has mainly focused on pressure, flow rate and efficiency. Among these, pressure difference and flow rate are in an inverse relationship and have been studied extensively. Changing the dimension of the micro-channel, and using multistage cascading and paralleling can increase the pressure difference or flow rate. The comprehensive performance of KP is mainly reflected in the efficiency. However, there is a serious lack of research on efficiency and the relevant theories are incomplete. This issue should be paid attention to in future research.
5. In the past 10 years, the applications of KP have developed rapidly. Based on the different properties reflected in the applications, the applications of KPs can be classified into three main categories: vacuum generation, flow delivery, and gas separation. KPs are expected to be applied in bioengineering, microchemistry, medical diagnosis and analysis, power engines, and other fields and devices.
